# Innovations in Nuclear Medicine Imaging for Reactive Oxygen Species: Applications and Radiopharmaceuticals

**DOI:** 10.3390/antiox13101254

**Published:** 2024-10-17

**Authors:** Joo Yeon Park, Sun Mi Park, Tae Sup Lee, Sang Ju Lee, Ji-Young Kim, Seung Jun Oh, Hai-Jeon Yoon, Bom Sahn Kim, Byung Seok Moon

**Affiliations:** 1 Department of Nuclear Medicine, Ewha Womans University Seoul Hospital, Ewha Womans University College of Medicine, Seoul 07804, Republic of Korea; jooy259@naver.com (J.Y.P.); psm9728@ewha.ac.kr (S.M.P.); 2 Division of RI Applications, Korea Institute Radiological and Medical Sciences, Seoul 01812, Republic of Korea; nobelcow@kirams.re.kr; 3 Department of Nuclear Medicine, Asan Medical Center, University of Ulsan College of Medicine, Seoul 05505, Republic of Korea; atlas425@amc.seoul.kr (S.J.L.); sjoh@amc.seoul.kr (S.J.O.); 4 Department of Nuclear Medicine, Ewha Womans University Mokdong Hospital, Ewha Womans University College of Medicine, Seoul 07985, Republic of Korea; gotothekorea@gmail.com (J.-Y.K.); haijeon.yoon@ewha.ac.kr (H.-J.Y.)

**Keywords:** reactive oxygen species, nuclear medicine, radiopharmaceutical, positron emission tomography, single photon emission computed tomography, oxidative stress

## Abstract

Reactive oxygen species (ROS) are generated during normal cellular energy production and play a critical role in maintaining cellular function. However, excessive ROS can damage cells and tissues, contributing to the development of diseases such as cardiovascular, inflammatory, and neurodegenerative disorders. This review explores the potential of nuclear medicine imaging techniques for detecting ROS and evaluates various radiopharmaceuticals used in these applications. Radiopharmaceuticals, which are drugs labeled with radionuclides, can bind to specific biomarkers, facilitating their identification in vivo using nuclear medicine equipment, i.e., positron emission tomography and single photon emission computed tomography, for diagnostic purposes. This review includes a comprehensive search of PubMed, covering radiopharmaceuticals such as analogs of fluorescent probes and antioxidant vitamin C, and biomarkers targeting mitochondrial complex I or cystine/glutamate transporter.

## 1. Introduction

Reactive oxygen species (ROS) are highly reactive molecules derived from oxygen, including perchloric acid, peroxide, superoxide, singlet oxygen, alpha oxygen, and hydroxyl radicals. These species are characterized by their property to easily lose or gain electrons, which contributes to their reactivity [1]. The primary source of ROS in cells is the mitochondria, where they are generated during oxidative phosphorylation, the process by which mitochondria produce ATP [1,2,3]. While ROS play an essential role in cellular signaling pathways, such as those involving nuclear factor kappa B and mitogen-activated protein kinase, only small amounts are required for their functions. Excess ROS are considered toxic by-products and are mitigated by cellular antioxidant systems, which include enzymes such as superoxide dismutase and small molecules such as vitamin C [1,2,4].

Residual ROS, which are not neutralized by antioxidants, can cause damage to proteins, lipids, and DNA, leading to various diseases, including cardiovascular, inflammatory, and neurodegenerative disorders [5,6,7]. This has led to growing interest in ROS as a biomarker for disease [6,7,8,9,10,11].

Traditionally, fluorescent probes have been employed for imaging ROS in living cells or tissue sections due to their simplicity and real-time imaging capabilities [12,13,14,15,16,17,18,19,20,21]. Common probes include dihydroethidium (DHE) and MitoSOX for superoxide (O_2_^•−^), Amplex Red and OxyR for hydrogen peroxide (H_2_O_2_), and dichloro-dihydro-fluorescein diacetate for general ROS detection [12,14,16,17,18,19]. However, these probes have limitations in tissue penetration and target-to-background ratio, restricting their use in vivo. 

Advanced techniques, such as electron paramagnetic resonance (also known as electron spin resonance) and luciferin-based peroxy-caged luciferin, for fluorescence imaging targeting ROS have been developed. Nevertheless, these methods face challenges that hinder widespread adoption, including the toxicity of paramagnetic probes and difficulties in clinical studies with fluorescent probes [1,21,22,23,24,25,26,27,28,29]. Nuclear medicine imaging, which involves labeling molecules, also called radiopharmaceuticals, that bind to in vivo biomarkers or exhibit similar behavior to biomarkers with radioactive isotopes, offers a promising alternative. This method allows for visualizing and quantifying biomarkers using methods such as positron emission tomography (PET) or single photon emission computed tomography (SPECT) [22,30,31,32,33,34,35,36,37,38]. 

Despite the progress in developing ROS-specific radiopharmaceuticals, their optimization for commercial use remains a critical area of ongoing research. Given the significant role of ROS in various diseases, non-invasive in vivo detection methods are crucial for advancing disease diagnosis and understanding disease mechanisms. Therefore, this review focuses on nuclear medicine imaging for ROS detection and discusses the radiopharmaceuticals available for ROS detection and their applications.

## 2. Radiopharmaceuticals for ROS Imaging

**Figure 1:**
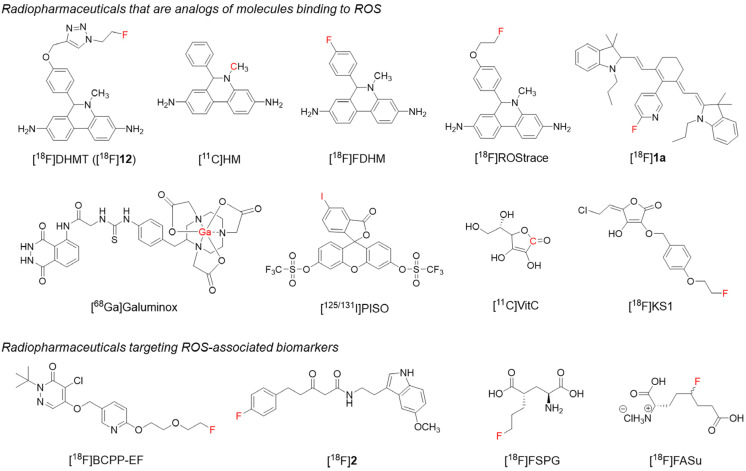
Chemical structures for radiopharmaceuticals targeting reactive oxygen species (ROS) and ROS-associated biomarkers. The red-highlighted elements represent the radioisotopes.

Radiopharmaceuticals have played an essential role in advancing ROS imaging and provide valuable tools for diagnosing and understanding various diseases. This section details the development and application of radiopharmaceuticals specifically designed for ROS imaging (Figure 1). 

A literature search was performed on PubMed (https://pubmed.ncbi.nlm.nih.gov/) by two authors (J.Y.P. and S.M.P.) in May 2024 to identify relevant articles. The search utilized the keywords “reactive oxygen species, radiopharmaceutical,” “reactive oxygen species, positron emission tomography,” and “reactive oxygen species, single photon emission computed tomography,” with no restrictions on the publication date. The inclusion criteria were (i) preclinical and clinical studies and (ii) research involving in vivo ROS imaging or the detection of biomarkers correlated with ROS levels. Exclusion criteria encompassed (i) reviews, meta-analyses, and conference abstracts; (ii) non-English publications; (iii) duplicate articles; (iv) inaccessible articles; and (v) studies not directly related to the research purposes. Various radiopharmaceuticals have been proposed for ROS imaging, and numerous studies have been conducted on this objective (Table 1). 

### 2.1. Radiopharmaceuticals That Are Analogs of ROS-Binding Molecules

#### 2.1.1. Analogs of Fluorescent Probes

Radiopharmaceuticals developed from labeling radioisotopes on fluorescent probes have shown promise in ROS imaging. Examples include DHE, hydrocyanine, luminol, and HKSOX-1 analogs.

##### Dihydroethidium

DHE is widely used for detecting superoxide due to its ability to permeate cell membranes and the blood-brain barrier (BBB) [39,53]. Upon oxidation by superoxide, DHE forms a positively charged product, 2-OH ethidium, which binds to DNA, providing long-term intracellular retention [54]. On this basis, several [^18^F]DHE (fluorine-18: β^+^ decay, half-life = 109.8 min) derivatives have been developed for ROS PET imaging.

**Figure 2:**
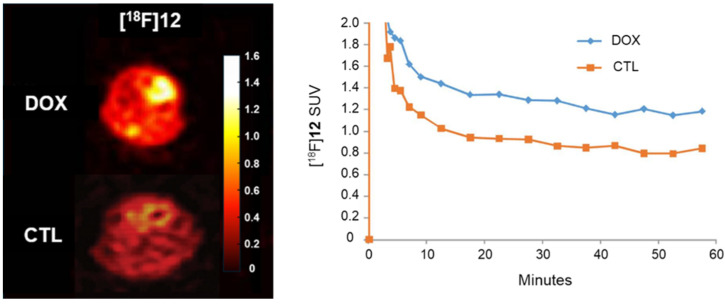
PET imaging of the heart in untreated (CTL) and DOX-treated (DOX) mice with [^18^F]**12**. Initial radioactive uptake in the heart was similar between the two groups, but after 1 h, radioactive uptake in DOX-treated mice was approximately 2-fold higher than in untreated mice. The images (**left**) show PET scans of the heart, with corresponding quantitative data (**right**) illustrating the difference in uptake over time. PET, positron emission tomography; DOX, doxorubicin. Reproduced from [39], copyright © 2014, Organic & Biomolecular Chemistry.

In 2014, Chu et al. [39] reported the development of [^18^F]**12**, a radiopharmaceutical suitable for PET imaging. Similar to DHE, compound **12**, the non-radioactive standard of [^18^F]**12**, exhibits minimal fluorescence before oxidation but demonstrates strong fluorescence at 595 nm upon oxidation, reacting selectively with superoxide. In experiments with the murine mammary carcinoma (EMT6) cell line treated with doxorubicin (DOX), compound **12** showed significantly increased fluorescence intensity compared with that of the control group. Moreover, in DOX-treated mice, [^18^F]**12** displayed twice the absorption relative to that of the control group, as confirmed by imaging (Figure 2). 

**Figure 3:**
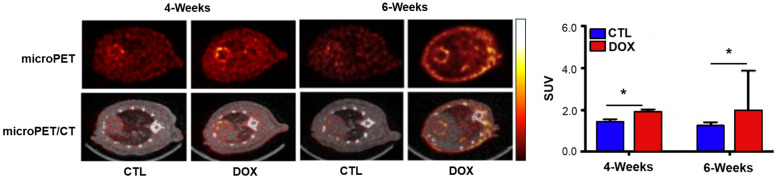
Quantitative evaluation of myocardial ROS activity using [^18^F]DHMT PET images. [^18^F]DHMT PET imaging represents microPET (**left**, **top**) and microPET/CT (**left**, **bottom**), with images from the control group (CTL) and DOX-treated mice (DOX) obtained at different time points (weeks 4 and 6). The bar graph (**right**) shows the comparative analysis of radioactive uptake between the groups (myocardial/blood SUV). PET, positron emission tomography; DOX, doxorubicin; ROS, reactive oxygen species; CT, computed tomography; SUV, standardized uptake value. * *p* < 0.05. Reproduced from [40], copyright © 2018, JACC: Basic to Translational Science.

[^18^F]**12**, also referred to as [^18^F]DHMT ([^18^F]6-(4-((1-(2-fluoroethyl)-1*H*-1,2,3-triazol-4-yl)methoxy)phenyl)-5-methyl-5,6-dihydrophenanthridine-3,8-diamine), has been indicated for the early detection of DOX-induced cardiotoxicity [40,41,42]. Using [^18^F]DHMT and PET imaging, early-stage cardiotoxicity could be detected in a DOX-treated rat model by assessing the left ventricular ejection fraction (LVEF) (Figure 3) [40]. Wu et al. [41] extended this study to larger animals, demonstrating the feasibility of the early detection of chronic DOX-induced cardiotoxicity using dynamic PET imaging in six beagle dogs. 

For mass production and clinical application, optimizing the automatic synthesis of radiopharmaceuticals is essential. Zhang et al. [42] successfully optimized an automated synthesis method for [^18^F]DHMT ([^18^F]**12**), achieving a moderate radiochemical yield of 6.9 ± 2.8% within a reduced preparation time of 77 min. Automated synthesis also demonstrated effective ROS imaging with a high myocardial-to-background ratio in PET imaging using [^18^F]DHMT in healthy beagle dogs.

**Figure 4:**
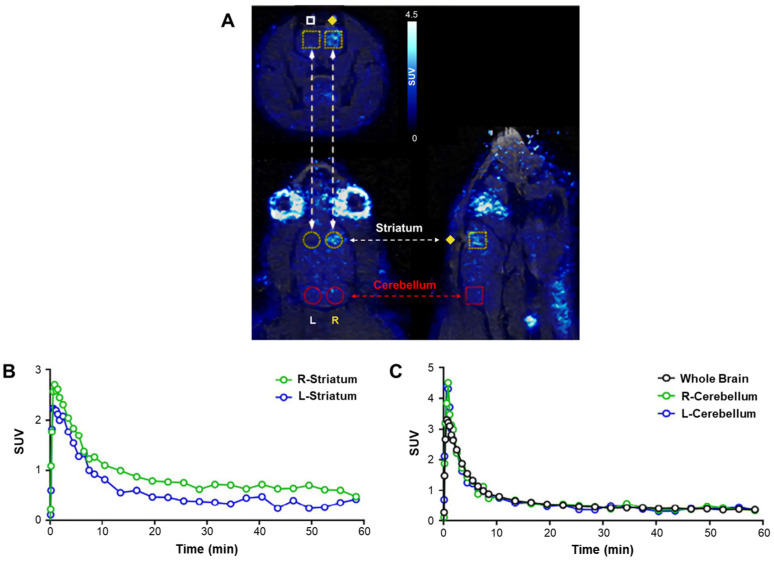
[^11^C]HM PET imaging. [^11^C]HM PET static image ((**A**), 0–60 min), including transverse, coronal, and sagittal views, showing cylindrical regions of interest (ROIs) representing the right (◆) and left (□) striatum and cerebellum. Time-activity curves of the right and left striatum (**B**) and the whole brain, right cerebellum, and left cerebellum (**C**) are shown. PET, positron emission tomography. Reproduced from [43], copyright © 2017, Nuclear Medicine and Biology.

ROS in the central nervous system have been implicated in neurodegenerative diseases. For imaging the central nervous system, radiopharmaceuticals must be able to specifically pass through the BBB. Several radiopharmaceuticals meeting this requirement have been developed. In 2017, Wilson et al. [43] developed and evaluated [^11^C]HM ([^11^C]Hydromethidine; carbon-11: β^+^ decay, half-life = 20.4 min) for ROS imaging in the central nervous system. PET images using [^11^C]HM showed increased signals in mice treated with sodium nitroprusside for ROS induction (Figure 4). The distribution and intensity of radioactive signals accurately reflect ROS levels in the brain. However, ^11^C-labeled derivatives present significant challenges for their practical application in clinical applications due to their short half-life of 20 min. This constraint complicates their practical use, as it requires specialized infrastructure for on-site production and immediate administration to patients.

**Figure 5:**
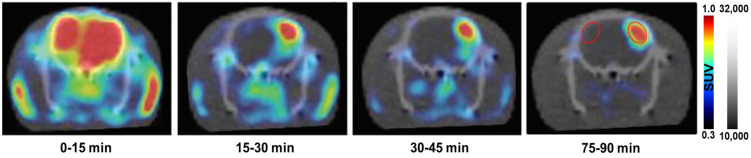
[^18^F]FDHM PET imaging of the brain. PET imaging was performed as dynamic images over 90 min, with image data integrated every 15 min. The red circles in the 75–90-min image indicate the area where SNP/saline was injected. PET, positron emission tomography; SNP, sodium nitroprusside. Reproduced from [44], copyright © 2020, Organic & Biomolecular Chemistry.

**Figure 6:**
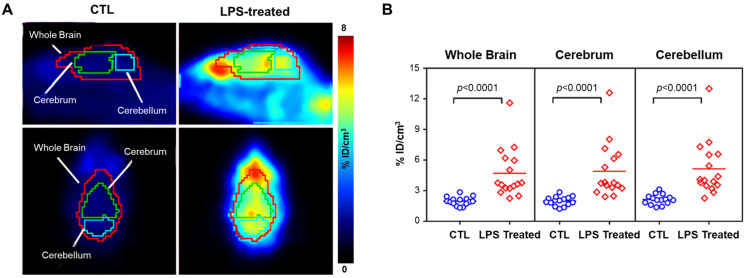
[^18^F]ROStrace PET imaging. Dynamic PET images were acquired for 60 min after intravenous [^18^F]ROStrace administration. (**A**) Representative image showing brain uptake from 40 to 60 min post-injection. (**B**) The graph compares the average %ID/cm^3^ values from 40 to 60 min post-injection between control and LPS-treated animals, showing a significant increase in the LPS-treated group. PET, positron emission tomography; LPS, lipopolysaccharide. %ID/cm^3^, percent injected dose per cubic centimeter. Reproduced from [45], copyright © 2018, ACS Chemical Neuroscience.

Egami et al. [44] reported a new PET radiotracer, ^18^F-labeled dihydromethidine ([^18^F]FDHM), for imaging ROS in the central nervous system (Figure 5). Their study demonstrated that [^18^F]FDHM can visualize ROS production in specific brain regions of living rats treated with sodium nitroprusside. [^18^F]FDHM was shown to pass through the BBB and was trapped in cells after being oxidized by ROS. In 2018, Hou et al. [45] reported [^18^F]ROStrace, another DHE analog. In a mouse model of lipopolysaccharide (LPS)-induced neuroinflammation, [^18^F]ROStrace showed high brain accumulation, enabling quantification and imaging of neuroinflammation (Figure 6). 

Weng et al. [20] further validated [^18^F]ROStrace for imaging superoxide levels in a model of LPS-induced neuroinflammation. [^18^F]ROStrace selectively reacts with superoxide and is converted to [^18^F]ox-ROStrace, which cannot pass through the BBB, indicating that superoxide can be selectively measured in the brain. The selectivity of DHE for superoxide and the characteristics of its absorption process are particularly useful. Notably, the ability of DHE analogs to cross the BBB, which is essential for imaging the central nervous system, represents a significant advantage. 

##### Hydrocyanine

Hydrocyanine, a probe that detects ROS at nanomolar concentrations [55,56], is a reduced cyanine dye structure activated by ROS. Initially, its structure is disrupted, leading to weak fluorescence due to broken π conjugation. Upon oxidation by superoxide and hydroxyl radicals, π conjugation is regenerated, significantly enhancing the fluorescence intensity by approximately 100-fold (*λ*_ex_ = 675 nm, *λ*_em_ = 693 nm). Compared with DHE, hydrocyanine exhibits excellent stability against autoxidation, even in a dissolved state, providing more reliable results.

**Figure 7:**
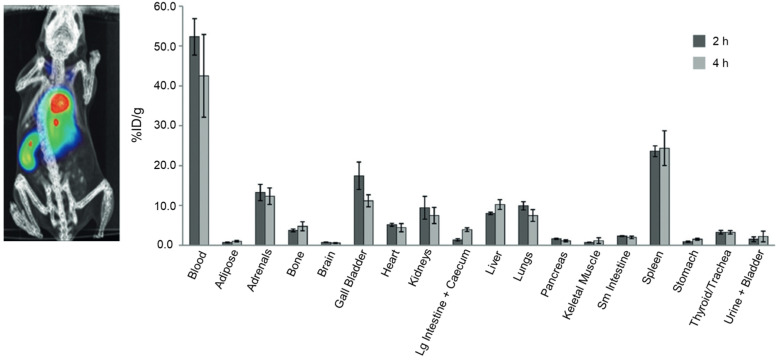
[^18^F]**1a** PET/CT image (**left**) and biodistribution results (**right**). [^18^F]**1a** PET image was obtained 2 h post-injection. Biodistribution data are expressed as percent injected dose per gram (% ID/g). PET, positron emission tomography; CT, computed tomography. Reproduced from [46], copyright © 2017, Chemistry.

Al-Karmi et al. [46] synthesized an ^18^F-labeled fluorinated hydrocyanine dye, [^18^F]**1a** (Figure 7), which is a probe capable of both PET and fluorescence detection. [^18^F]**1a** correlated with ROS detection via fluorescence in human prostate cancer (PC-3 cell line). The stability of [^18^F]**1a** against autoxidation has been confirmed in dynamic PET imaging. [^18^F]**1a** PET/CT imaging showed high blood pool radioactivity, visualizing blood-rich organs such as the heart, lungs, and spleen. Non-oxidized [^18^F]**1a** maintained stability, whereas the oxidized form showed reduced blood and spleen concentrations. [^18^F]**1a** is a notable radiopharmaceutical as it maintains the advantages of a fluorescent probe, is capable of PET imaging, and exhibits enhanced stability, demonstrating promise in terms of efficacy and safety. However, additional preclinical studies in disease models are necessary to further characterize the compound and confirm its potential for clinical applications.

##### Luminol

Luminol oxidizes and emits chemiluminescence at wavelengths of up to 425 nm and is among the most widely used chemiluminescent compounds due to its availability and low cost [57,58]. H_2_O_2_ significantly contributes to the oxidation and chemiluminescence of luminol. Due to these characteristics, luminol is used as a probe to detect neutrophil oxidation and is gaining attention in oxidative stress studies [47,58].

**Figure 8:**
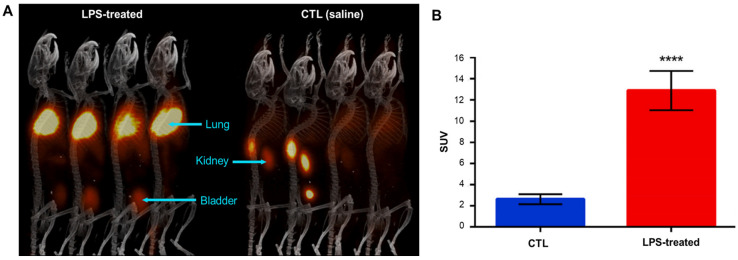
[^68^Ga]Galuminox PET/CT imaging. (**A**) PET images were acquired as 0–60-min dynamic scans; the displayed images are the 45–60-min summation frame. C57BL/6 mice in each group were intraperitoneally administered LPS (5 μg/g, 24 h after treatment, left) or saline (right). (**B**) SUV in the lungs of mice administered LPS or saline (mean ± SEM, **** *p* < 0.0001). The group administered LPS maintained a higher uptake value. PET, positron emission tomography; CT, computed tomography; SUV, standardized uptake value; LPS, lipopolysaccharide. Reproduced from [47], copyright © 2020, Redox Biology.

In 2020, an analog of luminol labeled with ^68^Ga (gallium-68: β^+^ decay, half-life = 67.8 min), [^68^Ga]Galuminox, was reported to detect superoxide and H_2_O_2_ production (Figure 8). [^68^Ga]Galuminox can be detected using both fluorescence and PET imaging methods. In in vitro studies, [^68^Ga]Galuminox demonstrated the ability to detect and monitor superoxide in LPS-induced A549 cells. The region marked by [^68^Ga]Galuminox fluorescence corresponded to the same position marked by MitoSOX. Dynamic PET/CT scans showed that [^68^Ga]Galuminox had 4-fold higher uptake and stable retention in the lungs of LPS-treated mice than in normal mice. Isolated lungs from these mice were tested for ROS using CellROX, a ROS probe, and the results showed a consistent pattern [47]. These data suggest that [^68^Ga]Galuminox uptake can be used as a measure of ROS activity in severely impaired lungs. Moreover, [^68^Ga]Galuminox is radiolabeled non-covalently, which simplifies its preparation compared to fluorine-18 derivatives. This feature indeed enhances its potential for easy in-house radiolabeling, making it more accessible for nuclear medicine practice. Further studies are needed to determine its applicability in other diseases.

##### HKSOX-1

The fluorescence principle of the HKSOX-1 probe involves superoxide cleavage of an aryl trifluoromethane sulfonate group to yield a free phenol [59]. Through this strategy, HKSOX-1 exhibited specific data even in the presence of intracellular reducing agents such as glutathione and was stable under various pH conditions (*λ*_ex_ = 509 nm, *λ*_em_ = 534 nm).

**Figure 9:**
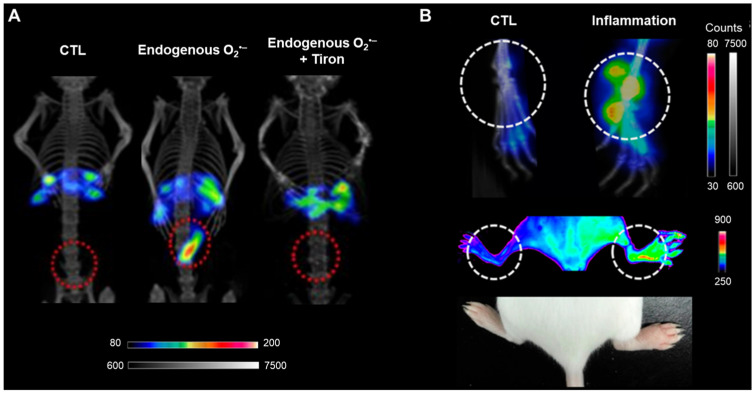
[^125/131^I]PISO SPECT/CT imaging. (**A**) [^131^I]PISO SPECT/CT images of an endogenous O_2_^•−^ model. Increased [^131^I]PISO uptake in the endogenous O_2_^•−^ model was reduced by Tiron (0.4 μg/g). (**B**) SPECT/CT images of an inflammation model following the intravenous injection of [^125^I]PISO (upper); fluorescence image of an inflammation mouse model 1 h after intravenous injection of 100 μL of PISO (1 mg/mL, middle); an image of the inflammation mouse model (lower). SPECT, single photon emission computed tomography; CT, computed tomography. Reproduced from [48], copyright © 2018, Analytical Chemistry.

[^125/131^I]PISO, an analog of HKSOX-1, has been designed and reported (Figure 9) [48]. It has shown high sensitivity and selectivity for superoxide, similar to those of HKSOX-1. Additionally, its ^125^I (iodine-125: γ decay, half-life = 59.39 days) or ^131^I (iodine-131: β^−^ and γ decay, half-life = 8.06 days) labeling lends it to SPECT imaging. The efficacy of [^131^I]PISO and [^125^I]PISO has been confirmed in endogenous O_2_^•−^ mice and a hind paw inflammation model. The results showed a pattern proportional to the degree of endogenous O_2_^•−^ and inflammation induction. Notably, the SPECT images and fluorescence data of the hind paw inflammation model showed the same pattern. [^125/131^I]PISO can be imaged using SPECT, and unlike most existing ROS probes, it reacts to superoxide through a non-oxidizing mechanism. This allows it to react efficiently and stably with superoxide without interference from other ROS molecules or antioxidants. In addition, [^125/131^I]PISO is anticipated to be applied to various diseases, providing a versatile tool for biomedical research.

#### 2.1.2. Antioxidant Analogs—Vitamin C

In living organisms, several antioxidants that scavenge ROS to prevent the negative effects exist [48]. Ascorbic acid, widely known as vitamin C, is one of the essential antioxidants. It participates in regulating ROS levels and enhances the effects of other antioxidants, such as vitamin E [60,61]. Vitamin C is slowly absorbed into cells through the sodium-dependent vitamin C transporters 1 and 2, but when oxidized to dehydroascorbic acid (DHA), it is rapidly absorbed through glucose transporters 1, 3, and 4 [21].

**Figure 10:**
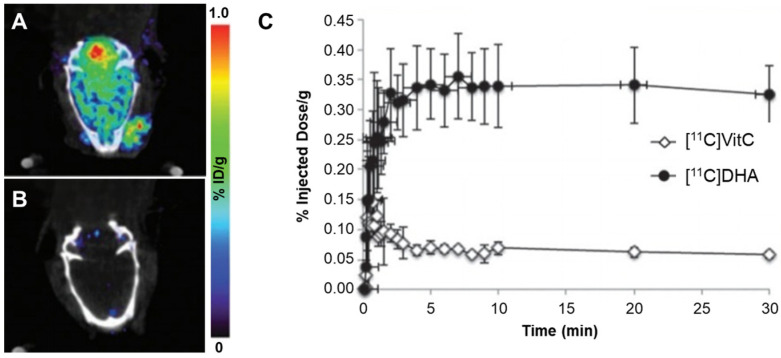
Representative PET images of [^11^C]DHA and [^11^C]VitC. (**A**) Oxidized form of [^11^C]VitC ([^11^C]DHA) and (**B**) [^11^C]VitC in a normal rat brain; (**C**) time-activity curve of the brain region of interest (ROI) data for dynamic scans. PET, positron emission tomography; DHA, dehydroascorbic acid Reproduced from [21], copyright © 2016, Chemical Communications.

Carroll et al. [21] reported [^11^C]ascorbic acid ([^11^C]VitC), an ascorbic acid derivative, as a radiopharmaceutical targeting ROS in the brain (Figure 10). In their study, [^11^C]VitC and its oxidized form, [^11^C]DHA ([^11^C]Dehydroascorbic acid), were administered to normal mice. The oxidized form showed significantly higher brain uptake than that of the non-oxidized [^11^C]VitC. These results indicated that [^11^C]VitC behaves similarly to ascorbic acid, allowing ROS expression levels in the brain to be confirmed through selective absorption depending on the presence or absence of [^11^C]VitC oxidation.

**Figure 11:**
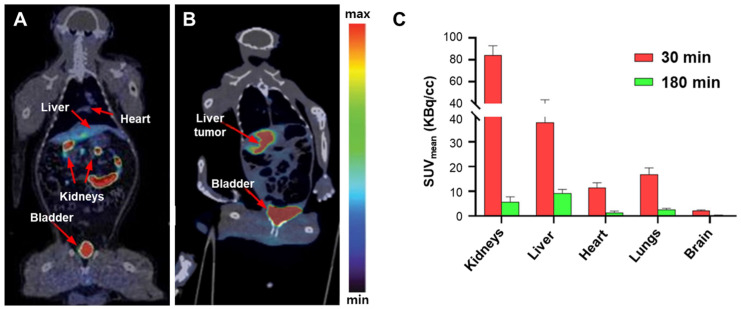
Representative PET/CT images of [^18^F]KS1. (**A**) A healthy rhesus monkey. (**B**) A monkey with an irradiated hepatic tumor. (**C**) Washout profile from 30 to 180 min in healthy rhesus monkeys. PET, positron emission tomography; CT, computed tomography. Reproduced from [30], copyright © 2022, Biomedicine & Pharmacotherapy.

Additionally, [^18^F]KS1 ([^18^F](E)-5-(2-chloroethylidene)-3-((4-(2-fluoroethoxy)benzyl)oxy)-4-hydroxyfuran-2(5*H*)-one), a fluoroethoxy furanose ring-containing ascorbate derivative, a radiopharmaceutical targeting tumor ROS, has been reported (Figure 11) [30,62]. [^18^F]KS1 has the advantage of a longer half-life compared with existing ^11^C-labeled radiopharmaceuticals. [^18^F]KS1 showed specific uptake at ROS expression sites in tumor-bearing and DOX-induced mice and tumor-bearing rhesus monkeys. When evaluating the pharmacokinetics and imaging efficiency of [^18^F]KS1 in healthy mice and rhesus monkeys, high accumulation in ROS-rich cells and clearance from major organs were observed. Both [^11^C]VitC and [^18^F]KS1 demonstrate specific ROS absorption. [^11^C]VitC is effective for imaging ROS within the brain, while [^18^F]KS1 is effective for imaging ROS expression in tumors. These radiopharmaceuticals provide valuable tools for studying ROS-related processes in different biological contexts.

### 2.2. Radiopharmaceuticals Targeting ROS-Associated Biomarkers

#### 2.2.1. Mitochondrial Complex I

Mitochondria are the primary source of ROS, with mitochondrial complex I (MC-I) serving as a major site for ROS generation [49]. ROS are produced during ATP synthesis via oxidative phosphorylation, and MC-I, the initial complex of the electron transport chain, is recognized as a significant contributor to this process. Consequently, MC-I has become a prominent target for radiopharmaceuticals aimed at ROS imaging [32,49,50].

##### Pyridazinone Derivatives

**Figure 12:**
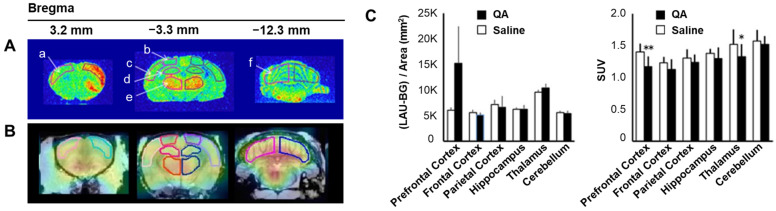
Detection of ROS production using DHE fluorescence and [^18^F]BCPP-EF PET imaging in QA-treated rat brain (**A**) Fluorescence accumulation in the quinolinic acid (QA)-injected striatum was observed 60 min after the intravenous injection of dihydroethidium (DHE). Regions of interest (ROIs) were identified as follows: prefrontal cortex (a), frontal cortex (b), parietal cortex (c), hippocampus (d), thalamus (e), and cerebellum (f). (**B**) [^18^F]BCPP-EF PET images were taken and summed 45–60 min post-injection. (**C**) Semiquantitative analysis of the fluorescent images (left) and radioactivity concentration measured as standardized uptake value (SUV, right) in QA and saline-treated rat brains. Data are presented as mean ± standard deviation. * *p* < 0.05, ** *p* < 0.01. PET, positron emission tomography. Reproduced from [49], copyright © 2021, EJNMMI Research.

Pyridazinone is a heterocyclic compound characterized by a six-membered ring known for its diverse pharmacological activities, including anti-inflammatory, analgesic, and antibacterial properties [63]. Hosoi et al. [49] investigated [^18^F]BCPP-EF (2-*tert*-butyl-4-chloro-5-{6-[2-(2[^18^F]fluoroethoxy)-ethoxy]-pyridin-3-ylmethoxy}-2*H*-pyridazin-3-one), a pyridazinone derivative, to understand ROS production and MC-I activity using PET imaging. In their study, quinolinic acid (QA) was injected into the striatum of rats, followed by DHE imaging and PET scans at 3 and 24 h post-injection. A strong DHE-induced fluorescence signal was observed in the striatum 3 h after QA injection. By 24 h, this signal had spread to other parts of the striatum and cerebral cortex. [^18^F]BCPP-EF uptake at 3 h after QA injection did not significantly change; however, a marked reduction was observed in the striatum and cerebral hemisphere after 24 h. Immunohistochemistry confirmed that fluorescent signals co-localized with microglial markers in the striatum after 24 h. The sensitivity of [^18^F]BCPP-EF PET was sufficient to detect the processes of QA-induced brain damage (Figure 12) [49]. Thus, [^18^F]BCPP-EF effectively demonstrated ROS production through MC-I activity and proved to be a valuable tool for imaging ROS within the brain. 

##### Curcumin/Melatonin Hybrid

**Figure 13:**
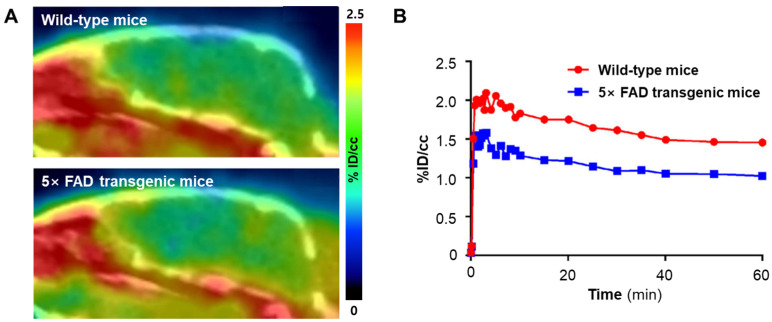
[^18^F]**2** PET imaging and time-activity curves of wild-type and 5× FAD mice. (**A**) [^18^F]**2** PET images of wild-type and 5× FAD mice, summed 20–60 min post-injection. (**B**) Time-activity curves represent the whole brain uptake of [^18^F]**2** in wild-type and 5× FAD mice. Data are presented as the mean percentage of the injected dose per cubic centimeter (% ID/cm^3^). PET, positron emission tomography. Reproduced from [50], copyright © 2021, ACS Chemical Neuroscience.

ZCM-I-1, a hybrid compound combining curcumin and melatonin, has demonstrated significant neuroprotective effects and is considered a potential therapeutic candidate for Alzheimer’s disease (AD). This hybrid compound substantially increases the expression levels of mitochondrial complexes I, II, and IV of the electron transport chain. Long-term treatment with ZCM-I-1 significantly reduced the levels of 8-hydroxyguanine, an indicator of oxidative damage to neuronal nucleic acids, and 4-hydroxy-2-nonenal, a lipid peroxidation marker. Moreover, ZCM-I-1 significantly decreased Aβ plaque accumulation in cortical and hippocampal regions in an APP/PS1 transgenic mouse model, thereby improving the pathological characteristics associated with AD [14,50,64,65]. Xu et al. [50] developed [^18^F]**2**, a radiopharmaceutical designed for MC-I imaging, based on the structural framework of ZCM-I-1. This novel PET radiotracer has shown neuroprotective effects by selectively binding to MC-I. In studies utilizing 5× FAD transgenic mice, an AD model, the brain radioactivity concentration was slightly reduced compared with that in wild-type mice. The PET tracer [^18^F]**2** has demonstrated potential in assessing MC-I dysfunction in the mouse model of AD (Figure 13) [50]. Therefore, [^18^F]**2**, through the PET imaging of MC-I activity, has exhibited its capability to evaluate MC-I dysfunction in AD mouse models, representing a promising tool for studying the progression and potential therapeutic interventions for AD.

#### 2.2.2. Cystine/Glutamate Transporter

The cystine/glutamate transporter, commonly referred to as system x_c_^−^, is a critical component of cell membranes responsible for the exchange of cystine and glutamate. This transporter facilitates cysteine uptake into cells, which is essential for the biosynthesis of glutathione, a potent antioxidant. Glutathione is vital in protecting cells from oxidative stress and safeguarding neuronal health in the brain. However, an excessive accumulation of glutamate can lead to excitotoxicity, which is detrimental to cell function and survival [51,66].

##### Glutamate

**Figure 14:**
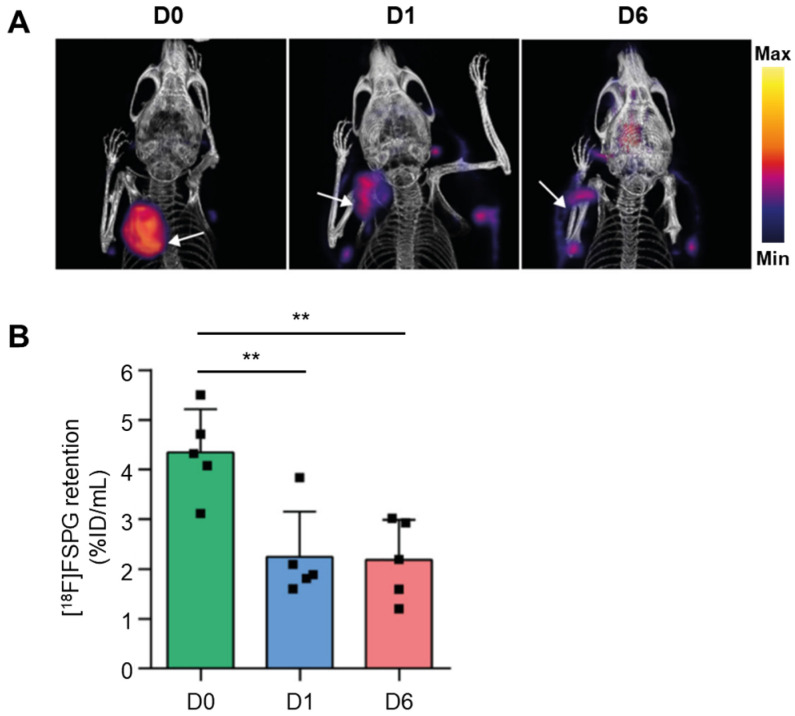
[^18^F]FSPG PET/CT imaging of A2780 ovarian cancer tumors in mice. (**A**) [^18^F]FSPG PET/CT images with tumors indicated by white arrows in three groups: untreated (D0), DOX-24 h (D1), or 6-day treatment (D6). (**B**) Quantified [^18^F]FSPG retention in the tumors of the three groups of mice. ** *p* < 0.01. PET, positron emission tomography; CT, computed tomography; DOX, doxorubicin. Reproduced from [51], copyright © 2019, Cancer Research.

McCormick et al. [51] identified [^18^F]FSPG ((S)-4-(3-[^18^F]fluoropropyl)-L-glutamic acid) as a radiopharmaceutical designed to evaluate the redox status of tumors. [^18^F]FSPG is internalized into the cell via system x_c_^−^, thereby delivering cystine, a crucial precursor for glutathione biosynthesis, which helps maintain the cellular redox balance. A study involving a mouse ovarian cancer model treated with the anticancer drug DOX showed a decrease in tumor [^18^F]FSPG uptake (Figure 14). This decrease corresponded to oxidative stress markers that appeared before observable changes in tumor volume. These findings suggest that [^18^F]FSPG PET imaging holds significant promise as an early predictor of tumor response to treatment. Additionally, it can potentially assess individual patient responses to therapy by monitoring antioxidant levels. This highlights the utility of [^18^F]FSPG PET in personalized medicine, where it could be employed to tailor treatments based on the antioxidant response of tumors, ultimately improving therapeutic outcomes.

##### L-Aminosuberic Acid

**Figure 15:**
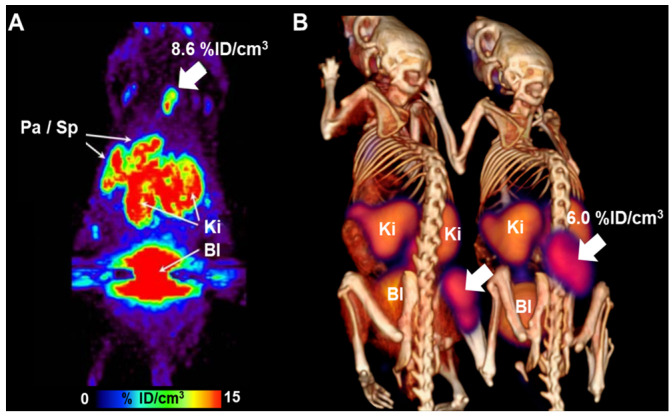
[^18^F]FASu PET imaging in mice bearing SKOV-3 and EL4 xenograft tumors. (**A**) The image shows the maximum-intensity projection of SKOV-3 tumor-bearing nude mice. (**B**) PET/CT image summed over 110–120 min after injection in Rag2 M mice bearing EL4 xenograft tumor. The tumor is indicated by a white arrow. PET/CT, positron emission tomography/computed tomography; Pa, pancreas; Sp, Spleen; Ki, Kidney; Bl, Bladder. Reproduced from [52], copyright © 2014, Journal of Nuclear Medicine.

L-Aminosuberic acid (L-ASu) is structurally more similar to L-cystine than to L-glutamate and is reportedly a more potent substrate for the system x_c_^−^ transporter. Webster et al. [52] developed L-ASu, incorporating the properties of both L-cystine and L-glutamate, and reported on [^18^F]FASu as a radiopharmaceutical for PET imaging. To evaluate the tumor uptake of [^18^F]FASu, SKOV-3 and EL4 tumors were implanted in mice for biodistribution and PET imaging studies. [^18^F]FASu acts as a substrate for system x_c_^−^, demonstrating high tumor uptake and prolonged retention in the mouse tumor model (Figure 15). Remarkably, [^18^F]FASu showed an absorption rate approximately 5-fold higher than that of [^18^F]FDG [52]. [^18^F]FASu emerged as a valuable metabolic radiopharmaceutical for PET imaging, enabling the visualization of cellular responses to oxidative stress. It may provide more sensitive detection than that by [^18^F]FDG in specific tumor types, such as ovarian adenocarcinoma (e.g., SKOV-3). Consequently, [^18^F]FASu also holds the potential for monitoring the response to anticancer treatment.

## 3. Discussion

ROS play a crucial role in various human diseases, including neurodegenerative, inflammatory, and cardiovascular diseases and tumors, underscoring the necessity of developing technology for in vivo ROS imaging [6,7,8,9,10,11]. This review aimed to summarize previous studies on nuclear medicine imaging for ROS detection and discuss available radiopharmaceuticals [21,39,40,41,42,43,44,45,46,47,48,49,51,56,62,66,67].

Radiopharmaceuticals targeting cardiotoxicity have been particularly noteworthy, especially as a side effect of anticancer drugs such as DOX [39,40,41,42,43,44,45,46,47,55,56,57,58,59]. Conventional diagnostic methods for cardiotoxicity include echocardiography and multigated acquisition scanning, which measure a decrease in LVEF [68,69]. However, these methods often detect cardiotoxicity only after significant myocardial damage has occurred, highlighting the urgent need for early diagnosis technologies [40,67,69]. Targeting ROS for early diagnosis, as demonstrated with [^18^F]DHMT, is promising since it can detect cardiotoxicity earlier than LVEF reduction [40]. Despite its higher cost relative to that of existing screening methods, the automatic synthesis of [^18^F]DHMT enhances its attractiveness for optimization and commercialization [42]. 

Brain PET imaging faces the significant challenge of the BBB, which restricts the delivery of radiopharmaceuticals to the brain [70]. However, derivatives such as [^18^F]ROStrace, [^18^F]FDHM, [^11^C]HM, and [^11^C]VitC have demonstrated the capability to cross the BBB and effectively image ROS in the brain [21,43,44,45]. Additionally, inflammation imaging in various body parts, including the lungs and ankles, has been successfully conducted with specific radiopharmaceuticals [56,58,59]. Furthermore, some research cases have identified tumors by exploiting the characteristic of ROS frequently occurring in tumors or by imaging the function of system x_c_^−^ [30,51,66]. Radiopharmaceuticals, e.g., [^18^F]BCPP-EF and [^18^F]**2**, have shown significant potential for imaging ROS and evaluating associated dysfunctions in diseases such as AD [49,50,63,64,65]. Advancements in these imaging technologies not only enhance our understanding of disease mechanisms but also improve early diagnosis and monitoring of therapeutic responses, ultimately contributing to personalized medicine approaches.

Despite these advances, several challenges to the widespread adoption of ROS-targeted radiopharmaceuticals remain. A significant limitation is the translation of preclinical results into clinical practice. Although several radiopharmaceuticals have shown promise in animal models, comprehensive validation of their efficacy and safety in humans is still lacking. Additionally, in vivo ROS imaging is inherently complex due to the transient and highly reactive nature of ROS [68]. Developing radiopharmaceuticals that can selectively and sensitively detect specific ROS species without significant off-target effects remains a critical area of research.

Furthermore, in the clinical translation of ROS-targeting radiopharmaceuticals, there are several key challenges that could be considered. First, as highlighted in this review, optimizing automated production systems is essential to supporting both nonclinical and clinical efficacy studies. Ensuring the stability of these radiopharmaceuticals also remains a critical concern, as most ROS-targeting compounds are sensitive to moisture and light. Developing stabilization strategies will be necessary to improve their robustness for clinical use. Furthermore, while it may be difficult to fully distinguish between radiopharmaceutical-generated and disease-generated ROS, the diagnostic doses typically used (in the mGy range) are expected to generate minimal ROS compared to levels associated with disease pathology. Another limitation is the relatively narrow target patient population for ROS imaging, which is currently focused on diseases with significant oxidative stress components (e.g., cancer, cardiovascular, or neurodegenerative disorders), which could limit the broader application of these radiopharmaceuticals. However, as the role of ROS in a wider range of pathologies becomes clearer, these limitations may diminish. Consequently, while these challenges are significant, ongoing advancements in radiopharmaceutical development and imaging technology hold the potential to overcome them and expand the clinical applicability of ROS imaging in the future.

## 4. Perspectives and Conclusions

ROS have emerged as an attractive biomarker for various diseases, and studies utilizing them are actively underway. Developing non-invasive imaging techniques to detect ROS in vivo is essential for advancing diagnostic and therapeutic strategies. In this review, we explored the potential of nuclear medicine imaging techniques for detecting ROS, highlighting various promising radiopharmaceuticals that have demonstrated significant potential in preclinical studies for imaging ROS-related processes across various diseases. Despite various existing challenges, continued research and innovation are essential to overcome these barriers and fully realize the clinical potential of ROS-targeted imaging ligands, ultimately enhancing disease diagnosis, monitoring, and personalized treatment approaches.

In conclusion, advances in radiopharmaceuticals for imaging ROS represent a significant leap forward in our ability to non-invasively diagnose and monitor a range of diseases. Despite the promising developments, there remain critical challenges to be addressed to effectively translate these innovations from the bench to bedside. Future research should focus on optimizing the selectivity and sensitivity of these radiopharmaceuticals to specific ROS types, ensuring minimal off-target effects, and improving safety profiles for clinical use. Furthermore, the integration of these imaging ligands into routine clinical practice will require extensive validation through large-scale clinical trials. The potential of these technologies to enhance personalized medicine by enabling earlier diagnosis and more accurate monitoring of therapeutic responses is enormous. As we continue to innovate and refine these tools, the collaboration between chemists, biologists, and clinicians will be pivotal in overcoming the existing hurdles. The ultimate goal is to develop robust, reliable, and widely accessible imaging technologies that can provide deeper insights into the role of ROS in disease pathophysiology, thereby improving patient outcomes across a variety of healthcare disciplines.

## Figures and Tables

**Table 1:** Features of the original articles reviewed.

Radiopharmaceuticals	Analog	Target	Target Region	Main Results	Ref.
[^18^F]DHMT ([^18^F]**12**)	DHE	superoxide	heart	In DOX-treated EMT6 cells, staining with compound **12** showed significantly higher fluorescence intensity than in the control group. Only cells treated with [^18^F]**12**, not its oxidized form, displayed uptake. DOX-treated mice also exhibited twice the uptake of [^18^F]**12** compared to control group.	[39]
				In rats with DOX-induced chronic cardiotoxicity, [^18^F]DHMT uptake was significantly higher than in the control group, enabling earlier diagnosis of cardiotoxicity than with conventional LVEF.	[40]
				The absorption pattern of [^18^F]DHMT was determined in beagle dogs, providing valuable insights into its biodistribution and pharmacokinetics.	[41]
				Automated synthesis for [^18^F]DHMT ([^18^F]**12**) was successfully optimized, achieving a moderate radiochemical yield of 6.9 ± 2.8% within a reduced preparation time of 77 min. Dynamic heart scans of a healthy beagle dog were performed to assess [^18^F]DHMT distribution and uptake.	[42]
[^11^C]HM			brain	[^11^C]HM uptake was observed after microinjecting SNP into one side of the rat brain, allowing for the distinction of specific brain regions with high ROS concentrations.	[43]
[^18^F]FDHM				[^18^F]FDHM uptake was observed after microinjecting SNP into one side of the rat brain. [^18^F]FDHM accumulated significantly in specific regions where SNP induced ROS.	[44]
[^18^F]ROStrace				[^18^F]ROStrace successfully passed through BBB in mice with brain nerve inflammation induced by LPS.	[45]
				By comparing DHE fluorescence imaging and ex vivo autoradiography images of [^18^F]ROStrace, a high correlation was observed between signal intensity and distribution in the two imaging modalities.	[20]
[^18^F]**1a**	hydrocyanin	superoxide, hydroxyl radical	blood pool	Dynamic scans confirmed that [^18^F]**1a** does not self-oxidize and is stable. Additionally, it is compatible with both fluorescence and PET imaging modalities.	[46]
[^68^Ga]Galuminox	luminol	superoxide, hydrogen peroxide	lung	The uptake of [^68^Ga]Galuminox was approximately 4-fold higher in the lungs of mice with LPS-induced inflammation than in control mice. Additionally, LPS-treated A549 cells showed fluorescence in the same regions for both Galuminox and MitoSOX.	[47]
[^125/131^I]I-PISO	HKSOX-1	superoxide	abdomen, ankle	In the abdomen of endogenous O_2_^•−^ mouse model, [^131^I]PISO PET demonstrated increased uptake compared to control subjects, but, this was significantly decreased upon superoxide removal by Tiron. Inflammation-induced ankles absorbed twice as much [^125^I]PISO as healthy ankles.	[48]
[^11^C]VitC	ascorbic acid	superoxide, hydrogen peroxide, hypochlorous acid	brain	[^11^C]VitC uptake in the rat brain was selective, depending on the presence or absence of oxidation.	[21]
[^18^F]KS1			tumor	In tumor-bearing rats, DOX-induced rats, and tumor-bearing rhesus monkeys, [^18^F]KS1 showed specific absorption at ROS expression sites.	[30]
[^18^F]BCPP-EF	pyridazinone	MC-I	brain	[^18^F]BCPP-EF PET scans were performed at 3 and 24 h after QA injection into the rat striatum to induce brain injury. No significant change in [^18^F]BCPP-EF uptake was observed at 3 h, but uptake in the striatum and cerebral hemisphere significantly decreased at 24 h.	[49]
[^18^F]**2**	curcumin/melatonin hybrid			In the comparison of [^18^F]**2** uptake in the brain between 5× FAD transgenic and wild-type mice, lower uptake was observed in AD mice.	[50]
[^18^F]FSPG	glutamate	system x_c_^−^	tumor	A2780 tumor-bearing mice treated with DOX were monitored using [^18^F]FSPG. Tumor retention of [^18^F]FSPG decreased by 42% 24 h after DOX treatment.	[51]
[^18^F]FASu	L-aminosuberic acid			The efficacy of [^18^F]FASu was compared to [^18^F]FDG in mice bearing SKOV-3 tumors. [^18^F]FASu exhibited a 5.2-fold higher tumor uptake and a 4.6-fold greater tumor-to-blood ratio than [^18^F]FDG.	[52]

DHE, dihydroethidium; DOX, doxorubicin; EMT6 cell, murine mammary carcinoma cell; LVEF, left ventricular ejection fraction; SNP, sodium nitroprusside; ROS, reactive oxygen species; BBB, blood-brain barrier; LPS, lipopolysaccharide; A549, human lung adenocarcinoma cell line; PET, positron emission tomography; MC-I, mitochondrial complex I; QA, quinolinic acid; AD, Alzheimer’s disease, system x_c_^−^, cystine/glutamate transporter; SKOV-3, human ovarian adenocarcinoma cell line.

## Data Availability

No data were used for the research described in the article.
